# Supplementary material for: Discovery and application of insertion-deletion (INDEL) polymorphisms for QTL mapping of early life-history traits in Atlantic salmon
Source: BMC Genomics. 2010 Mar 8;11:156. doi: 10.1186/1471-2164-11-156 (PMC2838853; doi:10.1186/1471-2164-11-156)
Supplement: Additional file 2 — Information on developed 76 locus single-run INDEL panel in Atlantic salmon. Information on fluorescence labeling, primer concentrations, PCR pooling and links to alignments, INDEL motifs and GENESCAN (Burge and Karlin 1997) predictions of genes/exons are available in html format. [file 1471-2164-11-156-S2.ZIP › Additionalfile2/snpsummary8604.html]

```
Cluster 2710 Contig 1

prev  Summary    Contig List  next
```

Size of Consensus sequence = 746

Number of sequences = 22

Minimum redundancy = 6

Key

A gi|29318210|gb|CB506564.1|CB506564 ssalmna006050 muscle\_skin Salmo salar cDNA, mRNA sequence  
B gi|117463799|gb|EG796018.1|EG796018 EST\_ssal\_evd\_15736 ssalevd thymus Salmo salar cDNA Salmo salar cDNA clone ssal\_evd\_520\_002\_rev 5', mRNA sequence  
C gi|117452228|gb|EG784447.1|EG784447 EST\_ssal\_evd\_40786 ssalevd thymus Salmo salar cDNA Salmo salar cDNA clone ssal\_evd\_554\_241\_rev 5', mRNA sequence  
D gi|29313258|gb|CB502032.1|CB502032 ssalra019095 head Salmo salar cDNA, mRNA sequence  
E gi|117439663|gb|EG771886.1|EG771886 EST\_ssal\_evd\_47481 ssalevd thymus Salmo salar cDNA Salmo salar cDNA clone ssal\_evd\_563\_311\_rev 5', mRNA sequence  
F gi|40541658|gb|BM413920.1|BM413920 EST00842 Atlantic salmon Lambda Zap Express testis cDNA library Salmo salar cDNA clone tsr\_003\_a03 5' similar to H2-K region expressed gene 2, NP\_034515, mRNA sequence  
G gi|76595851|gb|DV107522.1|DV107522 SGP283022 Atlantic salmon intestine SSH cDNA library Salmo salar cDNA clone ITO-0287, mRNA sequence  
H gi|117522115|gb|EG853842.1|EG853842 EST\_ssal\_eve\_14650 ssaleve thyroid Salmo salar cDNA Salmo salar cDNA clone ssal\_eve\_519\_343\_fwd 3', mRNA sequence  
I gi|117522116|gb|EG853843.1|EG853843 EST\_ssal\_eve\_14651 ssaleve thyroid Salmo salar cDNA Salmo salar cDNA clone ssal\_eve\_519\_343\_rev 5', mRNA sequence  
J gi|117439664|gb|EG771887.1|EG771887 EST\_ssal\_evd\_47482 ssalevd thymus Salmo salar cDNA Salmo salar cDNA clone ssal\_evd\_563\_311\_fwd 3', mRNA sequence  
K gi|117499625|gb|EG831842.1|EG831842 EST\_ssal\_eve\_10614 ssaleve thyroid Salmo salar cDNA Salmo salar cDNA clone ssal\_eve\_514\_151\_fwd 3', mRNA sequence  
L gi|117499636|gb|EG831853.1|EG831853 EST\_ssal\_eve\_10615 ssaleve thyroid Salmo salar cDNA Salmo salar cDNA clone ssal\_eve\_514\_151\_rev 5', mRNA sequence  
M gi|117451433|gb|EG783652.1|EG783652 EST\_ssal\_evd\_40072 ssalevd thymus Salmo salar cDNA Salmo salar cDNA clone ssal\_evd\_553\_248\_rev 5', mRNA sequence  
N gi|117451434|gb|EG783653.1|EG783653 EST\_ssal\_evd\_40073 ssalevd thymus Salmo salar cDNA Salmo salar cDNA clone ssal\_evd\_553\_248\_fwd 3', mRNA sequence  
O gi|89860936|gb|DY717059.1|DY717059 EST\_ssal\_rgb2\_72798 ssalrgb2 mixed\_tissue Salmo salar cDNA Salmo salar cDNA clone ssal\_rgb2\_617\_343\_fwd 3', mRNA sequence  
P gi|89860937|gb|DY717060.1|DY717060 EST\_ssal\_rgb2\_72799 ssalrgb2 mixed\_tissue Salmo salar cDNA Salmo salar cDNA clone ssal\_rgb2\_617\_343\_rev 5', mRNA sequence  
Q gi|117463798|gb|EG796017.1|EG796017 EST\_ssal\_evd\_15735 ssalevd thymus Salmo salar cDNA Salmo salar cDNA clone ssal\_evd\_520\_002\_fwd 3', mRNA sequence  
R gi|117452227|gb|EG784446.1|EG784446 EST\_ssal\_evd\_40785 ssalevd thymus Salmo salar cDNA Salmo salar cDNA clone ssal\_evd\_554\_241\_fwd 3', mRNA sequence  
S gi|117506830|gb|EG838589.1|EG838589 EST\_ssal\_eve\_1621 ssaleve thyroid Salmo salar cDNA Salmo salar cDNA clone ssal\_eve\_501\_105\_fwd 3', mRNA sequence  
T gi|117506831|gb|EG838590.1|EG838590 EST\_ssal\_eve\_1622 ssaleve thyroid Salmo salar cDNA Salmo salar cDNA clone ssal\_eve\_501\_105\_rev 5', mRNA sequence  
U gi|117474159|gb|EG806378.1|EG806378 EST\_ssal\_evd\_17745 ssalevd thymus Salmo salar cDNA Salmo salar cDNA clone ssal\_evd\_522\_276\_fwd 3', mRNA sequence  
V gi|117474170|gb|EG806389.1|EG806389 EST\_ssal\_evd\_17746 ssalevd thymus Salmo salar cDNA Salmo salar cDNA clone ssal\_evd\_522\_276\_rev 5', mRNA sequence

6 SNPs detected

A B C D E F G H I J K L M N O P Q R S T U V  cosegregation weighted

176 - - - - T . . T T T T T - - - - - - - - - -   6/6 90.91
177 - - - - G . . G G G G G - - - - - - - - - -   6/6 90.91
178 - - - - T . . T T T T T - - - - - - - - - -   6/6 90.91
179 - - - - G . . G G G G G - - - - - - - - - -   6/6 90.91
180 - - - - T . . T T T T T - - - - - - - - - -   6/6 90.91
181 - - - - G . . G G G G G - - - - - - - - - -   6/6 90.91
